# Malaria knowledge and experiences with community health workers among recently pregnant women in Malawi

**DOI:** 10.1186/s12936-020-03228-2

**Published:** 2020-04-15

**Authors:** Ashley Malpass, Jobiba Chinkhumba, Elizabeth Davlantes, John Munthali, Katherine Wright, Kathryn Ramsey, Peter Troell, Michael Kayange, Fannie Kachale, Don P. Mathanga, Dziko Chatata, Julie R. Gutman

**Affiliations:** 1 U.S. President’s Malaria Initiative, United States Agency for International Development (USAID), Lilongwe, Malawi; 2 University of Malawi College of Medicine, Malaria Alert Centre, Blantyre, Malawi; 3 Malaria Branch, Division of Parasitic Diseases and Malaria, Center for Global Health, Centers for Disease Control and Prevention (CDC), 1600 Clifton Rd. NE, Mailstop A06, Atlanta, GA 30322 USA; 4 Management Sciences for Health (MSH), Medford, MA USA; 5 U.S. President’s Malaria Initiative, Malaria Branch, Division of Parasitic Diseases and Malaria, Center for Global Health, Centers for Disease Control and Prevention, Lilongwe, Malawi; 6 Ministry of Health, Lilongwe, Malawi

**Keywords:** Malaria, Pregnancy, Community Health Workers, Malawi, Intermittent preventive treatment, Sulfadoxine-pyrimethamine

## Abstract

**Background:**

The World Health Organization recommends three or more doses of intermittent preventive treatment in pregnancy with sulfadoxine-pyrimethamine (IPTp-SP) to mitigate the negative effects of malaria in pregnancy (MIP). Many pregnant women in Malawi are not receiving the recommended number of doses. Community delivery of IPTp (cIPTp) is being piloted as a new approach to increase coverage. This survey assessed recently pregnant women’s knowledge of MIP and their experiences with community health workers (CHWs) prior to implementing cIPTp.

**Methods:**

Data were collected via a household survey in Ntcheu and Nkhata Bay Districts, Malawi, from women aged 16–49 years who had a pregnancy resulting in a live birth in the previous 12 months. Survey questions were primarily open response and utilized review of the woman’s health passport whenever possible. Analyses accounted for selection weighting and clustering at the health facility level and explored heterogeneity between districts.

**Results:**

A total of 370 women were interviewed. Women in both districts found their community health workers (CHWs) to be helpful (77.9%), but only 35.7% spoke with a CHW about antenatal care and 25.8% received assistance for malaria during their most recent pregnancy. A greater proportion of women in Nkhata Bay than Ntcheu reported receiving assistance with malaria from a CHW (42.7% vs 21.9%, p = 0.01); women in Nkhata Bay were more likely to cite IPTp-SP as a way to prevent MIP (41.0% vs 24.8%, p = 0.02) and were more likely to cite mosquito bites as the only way to spread malaria (70.6% vs 62.0% p = 0.03). Women in Nkhata Bay were more likely to receive 3 + doses of IPTp-SP (IPTp3) (59.2% vs 41.8%, p = 0.0002). Adequate knowledge was associated with increased odds of receiving IPTp3, although not statistically significantly so (adjusted odds ratio = 1.50, 95% confidence interval 0.97–2.32, p-value 0.066).

**Conclusions:**

Women reported positive experiences with CHWs, but there was not a focus on MIP. Women in Nkhata Bay were more likely to be assisted by a CHW, had better knowledge, and were more likely to receive IPTp3+ . Increasing CHW focus on the dangers of MIP and implementing cIPTp has the potential to increase IPTp coverage.

## Background

In sub-Saharan Africa, over 30 million pregnancies are exposed to *Plasmodium falciparum* transmission each year [1]. Of these, an estimated 10,000 pregnant women and up to 200,000 newborns die as a result of malaria in pregnancy. In addition, up to 8% of stillbirths globally are attributed to maternal malaria infection [2]. To mitigate the adverse effects of malaria in pregnancy, the World Health Organization (WHO) promotes the administration of intermittent preventive treatment in pregnancy (IPTp) with sulfadoxine-pyrimethamine (SP). Since 2012, the WHO has recommended that SP be administered as early as possible during the second trimester and at every scheduled antenatal clinic (ANC) visit thereafter, at least 1 month apart [3]. This recommendation followed a meta-analysis of seven studies that showed that receiving three or more doses of SP (IPTp3+) was associated with higher mean birth weights and less placental malaria than two doses of SP (IPTp2), with no differences in severe adverse events [4]. Despite this recommendation, progress with achieving IPTp3+ has been slow, and no sub-Saharan African country has reached the 85% target coverage of pregnant women for IPTp3+ , or even IPTp2+ [5].

Malawi was the first country to adopt IPTp-SP in 1993 [6], and has made significant progress in the number of women receiving IPTp2 since then, with coverage of 76.7% in 2017 [7]. However, only 40.1% of pregnant women received IPTp3+ in 2017, despite the fact that half of women attended four or more antenatal care (ANC) visits [8]. At the time of study initiation, Malawi was implementing the WHO Focused Antenatal Care (FANC) model, which recommends four ANC visits in pregnancy. This limits the number of opportunities for women to receive IPTp-SP. The Malawi Ministry of Health updated the ANC guidelines in June 2019 and now recommends eight ANC visits, in line with the 2016 WHO ANC model [9]. In addition, Malawi has implemented new registers to capture the additional visits.

Many factors contribute to low coverage of IPTp3+ , including barriers to ANC attendance, patient knowledge and attitudes, and facility level factors, such as SP availability, health worker performance, and poor documentation of SP doses [10]. Community delivery of IPTp-SP (cIPTp), which entails delivery of IPTp-SP to pregnant women by community health workers (CHWs), aims to increase women’s access to IPTp-SP [11–14].

This paper presents results of a pre-implementation baseline survey, highlighting recently pregnant women’s malaria knowledge, perceptions of CHWs, and barriers to care seeking, to better understand how cIPTp may impact IPTp coverage and ANC attendance.

## Methods

### Study location

**Fig. 1:**
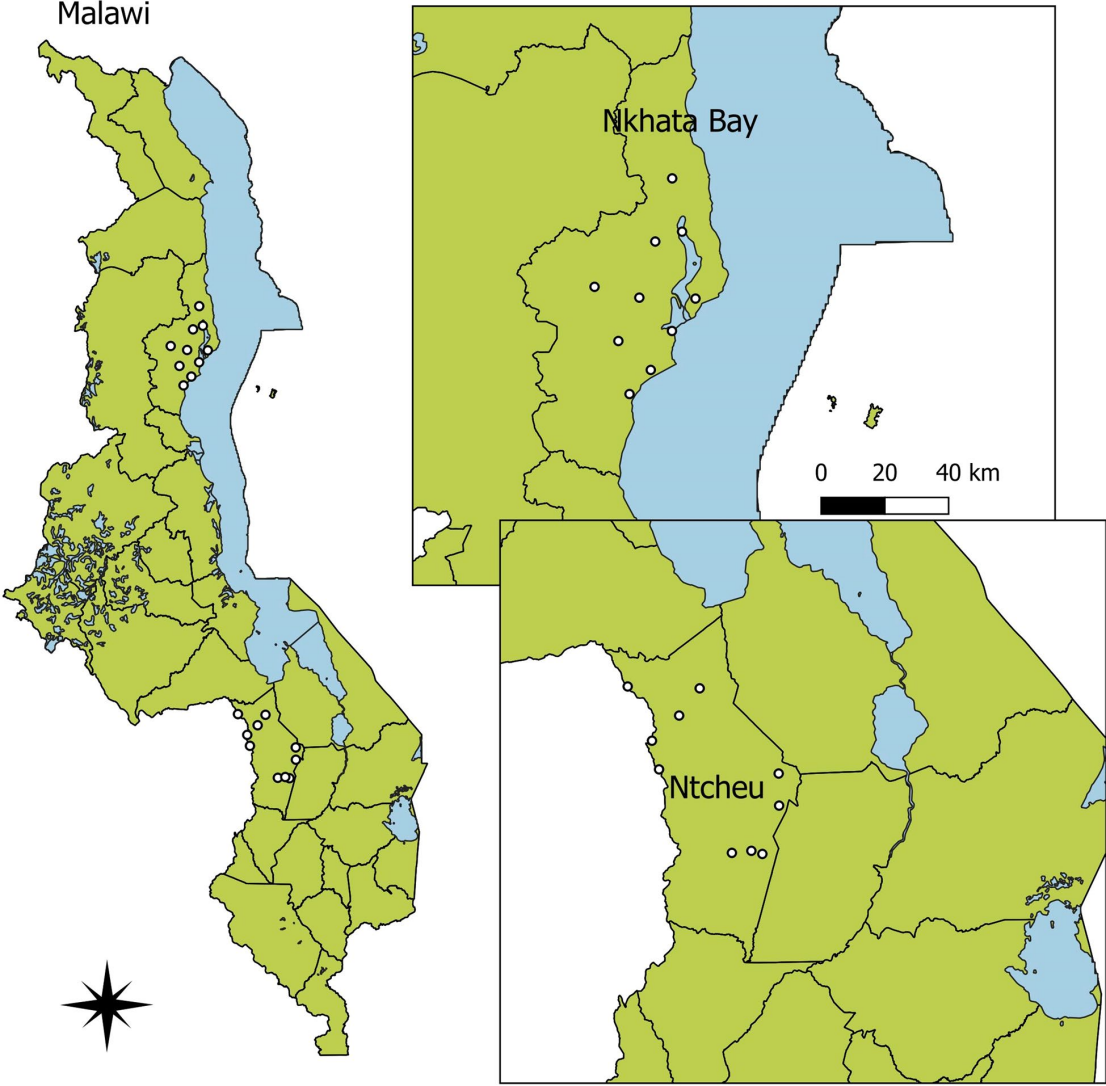
Map of the surveyed districts highlighting the locations of included health facilities

The survey was conducted in the districts of Ntcheu (pop—270,903), Central Region, and Nkhata Bay (pop—206,670), Northern Region, Malawi (Fig. 1). These districts were purposively selected from among the 10 districts in Malawi where the U.S. President’s Malaria Initiative (PMI) is supporting malaria control activities (out of 28 districts in Malawi) to ensure representation of two different regions. Both Ntcheu and Nkhata Bay are rural districts where most families survive on subsistence farming, with a small tourism economy serving Nkhata Bay district as well.

Ntcheu has almost twice as many health centres and ANC providers as Nkhata Bay (40 vs 22 and 55 vs 29, respectively), but only about 30% more pregnant women (13,544 vs 9905 annually) (Table 1). Nkhata Bay has more CHWs than Ntcheu; thus, Ntcheu has fewer CHWs per facility and each CHW, on average, serves a larger population. Although Ntcheu has fewer CHWs per facility, there are additional volunteers in the community called Secret Mothers. These volunteers receive minimal training and their primary function is to encourage women to attend ANC in the first trimester. The median age of marriage for women in both districts is 18 years old, while the median age at first birth is 19 years old. Women in both districts are equally likely to deliver at a health facility and with a nurse or midwife present [8].

**Table 1:** Characteristics of the study districts

District	Public health centres providing ANC	ANC staff	Number of CHWs	Total population	Pregnant population	IPTp2+ (%)^a^	IPTp3+ (%)^a^	ANC1+ (%)^a^	ANC4+ (%)^a^
Nkhata Bay	22	29	87	206,670	9905	65.4	31.3	98.1	57.6
Ntcheu	40	55	81	270,903	13,544	60.5	26.6	94.2	45.9

*ANC* antenatal care, *IPTp* intermittent preventive treatment in pregnancy

^a^National Statistical Office (NSO) [Malawi] and ICF. 2017. *Malawi Demographic and Health Survey 2015*–*2016*

Zomba, Malawi, and Rockville, Maryland, USA. NSO and ICF

### Population and sampling

The population of interest was recently pregnant women, defined as women between the ages of 16–49 years who had a pregnancy resulting in a live birth in the previous 12 months. A three-stage cluster sampling procedure was used to select survey respondents. After excluding District Hospitals, non-governmental facilities, facilities that do not provide ANC, and facilities accessible only by boat, 10 health centres were randomly selected in each district (20 in total). First, the catchment area of each health centre was designated as a cluster. Depending on geographical size, each cluster contained 3–18 enumeration areas (EAs). EAs are administrative data collection units, demarcated by the National Statistics Office, with an average of 250 households or 1000 people. In the second stage, a single EA was randomly selected from each cluster. Finally, all households within the selected EA were listed, noting households with recently pregnant women. Simple random selection was used to select 20 households with recently pregnant women and 20 households without recently pregnant women. If a selected household had more than one recently pregnant woman living in it, all women who met the inclusion criteria were included. EpiSample (PATH, MACEPA Developer Products, Seattle, WA) was used to map the households and sample participants.

### Sample size

It was estimated that a minimum of 300 recently pregnant women, 150 per arm, would be needed to achieve 80% power to detect a 30-percentage point increase in IPTp3 coverage from 30% at baseline to 60% at endline, with 0.05 significance level, assuming an intra-cluster correlation of 0.2, using a two-sided Z test (unpooled).

### Data collection

Twenty-eight (28) enumerators took part in the baseline household survey. All enumerators participated in a 2-day training followed by 1 day of piloting the survey in one of the non-selected EAs, and then a day of debriefing, during which minor updates were made to several questions to improve the readability and clarity.

The topics covered in the training included basic information about malaria and detailed instructions on how to map and administer the survey, including obtaining consent. The questionnaire was reviewed in both English and Chichewa (the local language) to ensure that the enumerators had good understanding of the overall organization, structure, and purpose of the questionnaire and to verify that the Chichewa questions were true reflections of their English counterparts. The survey was administered verbally as a structured questionnaire and questions were predominantly open response, allowing for multiple responses to be recorded. Whenever possible, ANC cards were used to validate women’s recall on the number of ANC visits attended and doses of SP received during their most recent pregnancy. Data were collected from December 1–16, 2017.

### Data management

Surveys were conducted using Open Data Kit (ODK) Forms hosted on the SurveyCTO (Dobility, Inc., Cambridge, MA) platform. Files from SurveyCTO were exported into SAS V9.4 (SAS, Cary, NC) and STATA (SATACorp, College Station, TX) for analysis. The database was programmed with range checks and key fields were required. Data were checked for completeness by field supervisors before uploading. In addition, data checks for key variables were run on data downloaded from the SurveyCTO platform.

### Analysis

Descriptive summary statistics including means, proportions, and corresponding 95% confidence intervals (CI) for social-demographic attributes, out of pocket expenditures, and time required for ANC attendance for women in the two districts were calculated. Households were grouped into wealth terciles based on reported monthly incomes; women with no monthly income were considered “poor” while women with any income were grouped into middle and high categories based on the median income. A malaria knowledge index was created to categorize women’s overall knowledge. Women were awarded one point for each of the following: correctly identifying (1) two or more health problems in pregnancy, (2) two or more consequences of malaria in pregnancy, (3) mosquito bites as the primary way to get malaria (with no incorrect methods, i.e., dirty water, identified), and (4) both IPTp and insecticide-treated nets (ITNs) as malaria prevention in pregnancy. Women with a score of 2 or less were categorized as having poor knowledge, while 3 or more was considered adequate knowledge. A logistic regression model using generalized estimating equations (proc genmod) accounting for the effects of district, age, gravidity, and facility-level clustering was used to assess the impact of knowledge on IPTp uptake. Statistical significance was assessed using t-tests for differences and means, and Chi square tests for proportions between the districts, for normally distributed data. Non-parametric tests were used for skewed data. P-values less than 0.05 were considered statistically significant. All analyses accounted for selection weight and clustering of households at facility level. Analyses were done using SAS V9.4 (Cary, NC). A comparison of the two districts was undertaken to explore heterogeneity between the districts.

### Ethics

The protocol was reviewed and approved by the Malawi College of Medicine Research Ethics Committee (COMREC); the Centers for Disease Control and Prevention Human Subjects Office determined that CDC staff were not engaged in human subjects research. Representatives from the Government of Malawi Ministry of Health were involved throughout the design and implementation of the survey, and permission was obtained from the officials in each District Health Office prior to initiating the survey. In each EA, permission was obtained from the village leaders. Written informed consent was obtained from each respondent before data collection; participants were told that this survey was focused on antenatal care and malaria prevention in pregnancy.

## Results

### Social demographic features

A total of 370 recently pregnant women responded to the survey; 179 in Nkhata Bay and 191 in Ntcheu. There were no refusals. Overall, the women in the two districts were similar across socio-demographic features, attitudes, perceptions, and ANC utilization. The median age of surveyed women was 23 (range: 16–44); 33.0% of respondents were primigravid, 25.6% were secundigravid, and 41.3% were multigravid. Only a quarter of women had received any secondary education; women in Nkhata Bay were more likely to have received secondary education than in Ntcheu (30.7% vs 21.5%, p = 0.05). The majority of women in both districts were considered poor based on household incomes (Table 2).

**Table 2:** Socio-demographic features

Indicator	Overall	Nkata Bay	Ntcheu	p-value
	N = 370	N = 179	N = 191	
Age in years (mean)	25 (16–44)	24.9 (16–43)	25.1 (17–44)	0.79
Gravidity				
Primi (%)	33.0	33.5	32.8	0.71
Secundi (%)	25.6	27.7	24.5	
Multi (%)	41.3	38.8	42.7	
Gravidity (mean)	2.5	2.5	2.5	0.92
Married (%)	83.9	81.3	84.5	0.32
Secondary education (%)	24.9	30.7	21.5	0.05
Wealth groups (%)				
Poor	65.4	60.7	66.8	0.19
Middle	17.1	17.0	17.2	
Least poor	17.4	22.3	16.0	

### Malaria in pregnancy knowledge

Malaria was the most frequent response given by recently pregnant women when asked to list serious problems in pregnancy (43.4%), with women in Nkhata Bay being more likely to consider it a serious problem (51.2% vs 41.2%, p = 0.02). Women in Nkhata Bay were also more likely to acknowledge maternal death and abortion/miscarriage as possible effects of malaria in pregnancy (55.4% vs 42.6%, p < 0.001 and 48.3% vs 29.1% p < 0.0001, respectively). A majority of women in both districts cited sleeping under an ITN as a way to prevent malaria, with no difference between districts (85.6% vs 85.2%, p = 0.92), while a minority cited IPTp, with women in Nkhata Bay more likely than women in Ntcheu to cite taking IPTp as a method of malaria prevention during pregnancy (41.0% vs 24.8%, p = 0.02) (Table 3).

**Table 3:** Knowledge of women about Malaria in Pregnancy

Indicator	Overall	Nkhata Bay	Ntcheu	p-value
	N = 369	N = 178	N = 191	
Serious problems during pregnancy (%)				
Accelerated/reduced fetal movement	2.1	2.1	2.0	0.97
Malaria	43.4	51.2	41.2	0.02
Convulsions	11.4	4.6	13.4	0.01
Loss of consciousness	2.9	3.2	2.8	0.87
Losing water	10.9	14.8	9.8	0.36
High fever	10.4	16.7	8.5	0.13
Bleeding	40.5	45.6	39.1	0.31
Severe weakness	2.5	5.4	1.7	0.10
Severe abdominal pain	9.2	11.7	8.5	0.46
Difficulty breathing	4.1	3.2	4.4	0.63
Severe headache	8.1	11.3	7.2	0.21
Swollen hands/face	16.0	18.9	15.1	0.49
Blurred vision	2.8	1.0	3.4	0.01
Signs of malaria (%)				
Fever	86.2	90.7	84.8	0.28
Diarrhoea	16.2	20.7	14.9	0.36
Vomiting	14.3	9.9	15.6	0.002
Convulsion	8.9	6.7	9.6	0.46
Aches and pains	42.7	47.3	41.3	0.36
How women can get malaria (%)				
Mosquito bites	79.6	89.9	76.6	< 0.0001
Dirty food/water	7.3	9.1	6.8	0.42
Dirty environment	13.7	21.7	11.3	0.005
Identified mosquito bites as the primary means to get malaria	68.6	62.1	70.6	0.03

	N = 355	N = 172	N = 185	
How to prevent malaria (%)				
Take IPTp	28.6	41.0	24.8	0.02
Sleep under ITN	85.3	85.7	85.2	0.92
Respondent identified both ITN and IPTp as prevention for MIP	18.7	29.3	15.5	< 0.0001
Respondent identified either ITN OR IPTp as prevention for MIP	90.1	93.6	89.1	0.002
Effects of malaria in pregnancy (%)				
Maternal death	45.5	55.4	42.6	0.0004
Abortion/miscarriage	33.4	48.3	29.1	< 0.0001
Baby might die or be dead at birth	32.3	38.9	30.3	0.09
Premature delivery	19.9	27.2	17.7	0.04
Severe maternal malaria	15.5	21.7	13.7	0.15
Baby might be small	11.4	12.8	11.0	0.59
Anemia	11.2	17.6	9.3	0.001

		N = 172	N = 183	
Knowledge index^a^				
Poor	66.2	63.9	66.9	0.64
Adequate	33.8	36.1	33.1	

^a^Knowledge index: A malaria knowledge index was created to categorize women’s overall knowledge. Women were awarded one point for each of the following: correctly identifying (1) two or more health problems in pregnancy, (2) two or more consequences of malaria in pregnancy, (3) mosquito bites as the only way to get malaria, and (4) both IPTp and ITNs as malaria prevention in pregnancy. Women with a score of 2 or less were categorized as having poor knowledge, while 3 or more was considered adequate knowledge

*IPTp* Intermittent preventive treatment in pregnancy, *ITN* Insecticide-treated Net, *MIP* malaria in pregnancy

Women in Nkhata Bay were more likely to cite mosquitos as a cause of malaria and to know that both ITNs and SP are a prevention strategy for malaria in pregnancy (89.9% vs 76.6% p < 0.001 and 29.3% vs 15.5% p < 0.0001, respectively) (Table 3). However, women in Ntcheu were more likely to know that mosquito bites are the only way to get malaria and less likely to attribute malaria to a dirty environment (70.6% vs 62.0% p = 0.03 and 11.3% vs 21.7% p = 0.005, respectively). Despite this, women in Ntcheu were not any less likely to report sleeping under a bed net every night compared to women in Nkhata Bay (94.8% in both districts, p = 0.99). Women’s malaria knowledge overall, based on the composite malaria score, was low and did not vary significantly between the two districts (only 36.1% and 33.1% had adequate knowledge, p = 0.64). Adjusting for district, mother’s age, and gravidity, women who had adequate knowledge were more likely to receive IPTp3 than women with poor knowledge, though this was not statistically significant (adjusted odds ratio = 1.50, 95% confidence interval 0.97–2.32, p-value 0.066)). A similar proportion of women in the two districts reported having had at least one case of malaria during their most recent pregnancy (36.3% vs 27.6%, p = 0.06) (Table 4).

**Table 4:** Antenatal Care (ANC) and Intermittent Preventive Treatment in Pregnancy (IPTp) Coverage

Indicator	Overall	Nkhata Bay	Ntcheu	p-value
	N = 276	N = 131	N = 145	
ANC attendance from health passport review				
ANC1	99.6	98.1	100.0	0.20
ANC2	97.5	94.0	98.6	
ANC3	83.8	74.5	86.6	
ANC4+	49.8	49.3	50.0	

	N = 297	N = 139	N = 158	
IPTp coverage from health passport review and self-report				
IPTp1+	88.8	89.5	88.6	0.85
IPTp2+	70.9	79.5	68.3	0.001
IPTp3+	45.8	59.2	41.8	0.0002
IPTp4+	15.8	20.9	14.3	0.26

	N = 357	N = 168	N = 189	
Timing of first ANC visit (self-report)				
0–12 weeks (less than 3 months)	15.6	10.2	17.1	0.0008
13–15 weeks (3 months)	27.5	14.6	31.1	
16–19 weeks (4 months)	27.2	26.1	27.6	
20–23 weeks (5 months)	15.7	24.0	13.3	
24–27 weeks (6 months)	10.3	19.1	7.8	
28–39 weeks (7–9 months)	3.2	6.1	2.8	

	N = 368	N = 177	N = 191	
First person told about the pregnancy				
Husband/partner	82.9	75.0	85.2	0.001
Mother	8.9	9.9	8.6	
Other family member - > grandmother	2.7	7.0	1.5	
Health facility worker	1.7	0.4	2.0	
Mother in law	1.4	2.7	1.1	
Sister in law	0.9	1.9	0.6	
Sister	0.7	2.6	0.1	
When that person was told				
0–3 months	94.4	93.6	94.6	0.86
4 months	2.5	3.3	2.3	
5–6 months	1.9	2.7	1.7	
7 months or more	0.3	0.4	0.3	

	N = 367	N = 177	N = 190	
Had malaria during recent pregnancy	29.6	36.3	27.6	0.06

Number of times (%)	N = 114	N = 66	N = 48	
1	65.3	58.0	68.1	0.13
2	22.7	31.5	19.3	
3+	12.0	10.5	12.6	

*ANC* Antenatal care

### Community health workers

The majority of recently pregnant women in both districts found the CHWs in their communities to be generally helpful (77.9%). In both districts, the most frequent responses as to why women liked the CHWs were because they provide good care and are easily available (46.4% and 32.2%, respectively), but more women in Nkhata Bay reported liking CHWs because they provide good care (60.3% vs 43.4% p = 0.02). In both districts, CHW accessibility and supply availability were the most commonly cited areas needing improvement (40.6% and 15.3%, respectively) (Table 5).

**Table 5:** Attitudes, perceptions, knowledge and exposure of pregnant women to community services provided by Community Health Workers (CHWs)

Indicator	Overall	Nkata Bay	Ncheu	p-value
	N = 370	N = 179	N = 191	
Median # CHWs (range)	0.8 (0–15)	1.3 (0–8)	0.7 (0–15)	0.009

	N = 306	N = 140	N = 166	
Reported number of CHWs in the village				
0 CHW	4.8	12.3	2.9	0.009
1 CHW	59.2	30.7	66.6	
2 CHWs	17.3	23.8	15.7	

	N = 278	N = 123	N = 155	
Reported female CHWs (%)	41.9	39.2	42.5	0.82

	N = 279	N = 123	N = 156	
Women that find CHWs helpful (%)	77.9	71.9	79.3	0.4

	N = 223	N = 89	N = 134	
What women like about CHWs (%)				
Gives good care	46.4	60.3	43.4	0.02
Easily available	32.2	24.3	33.9	0.26
Close to home	22.7	23.4	22.5	0.90
Understand my community	14.7	19.9	13.6	0.04
Nice personality	10.0	9.4	10.1	0.89
Not expensive	6.0	1.2	7.0	< 0.0001

	N = 280	N = 124	N = 156	
Wanted CHWs improvements (%)				
Improve CHWs accessibility	40.6	41.1	40.5	0.94
Improve supply availability	15.3	13.2	15.8	0.79
Improve village clinic time	12.1	21.2	10.0	0.04
Improve knowledge base	10.0	16.1	8.6	0.03
Improve village clinic location	7.3	14.4	5.7	0.03

	N = 280	N = 124	N = 156	
Malaria services provided by CHWs (%)				
Advice on using nets	50.7	60.3	48.4	0.03
Malaria treatment	15.2	12.1	15.9	0.44
Advice on seeking malaria treatment	11.2	12.4	10.9	0.78
Rapid test for malaria	8.8	10.1	8.5	0.79
Dispense drugs to prevent malaria	7.9	9.3	7.5	0.59
Referral to ANC	5.1	7.9	4.5	0.17
Advice on obtaining drugs to prevent malaria	4.7	2.4	5.3	0.19
None	17.4	12.8	18.4	0.39
Issues CHWs has helped with (%)				
Nothing	31.8	32.6	31.6	0.89
Malaria	25.8	42.7	21.9	0.01
Diarrhoea/vomiting	15.7	8.9	17.2	0.003
Water/sanitation	14.1	14.6	14.0	0.90
Nutrition	12.7	3.4	14.9	0.009
Vaccines/mass treatment	9.1	4.4	10.2	0.03
Pregnancy	8.5	9.1	8.3	0.83
Education	6.3	1.8	7.4	0.16
Family planning	6.2	14.4	4.3	< 0.0001
Pneumonia/lower respiratory tract infection	2.6	0.7	3.0	0.05
Muscle pains	0.5	1.3	0.4	0.25

*CHW* Community Health Worker, *ANC* antenatal care

Although few women (8.5%) mentioned discussing pregnancy as a health topic with the CHW in their community (Table 5), one-third of the women (35.7%) reported talking to a CHW during their most recent pregnancy. Among these, half (49.3%) talked to their CHW two or more times during their most recent pregnancy. In Ntcheu, conversations with CHWs were most likely to take place during a home visit (47.7% vs 19.9% p = 0.002), while women in Nkhata Bay were most likely to speak with CHWs at the health facility (47.5% vs 16.3% p = 0.02). Overall, the three main topics discussed during interactions with CHWs were: diet during pregnancy (38.6%), the plan for delivery (36.1%), and when to attend ANC (35.4%). Sleeping under an ITN was the most common advice given to pregnant women during CHW visits (92.0%); only 28% of women reported being advised by the CHW to take SP during pregnancy (Table 6). Less than one quarter (21.9%) of women in Ntcheu and 42.7% of women in Nkhata Bay reported that they or their family had received assistance from a CHW for malaria (p = 0.01) (Table 5).

**Table 6:** Interactions with Community Health Workers (CHWs) during most recent pregnancy

Indicator	Overall	Nkhata Bay	Ncheu	p-value
	N = 370	N = 179	N = 191	
Talked with CHWs about ANC or related topic (%)	35.7	30.5	37.2	0.25

	N = 123	N = 51	N = 72	
Frequency of interaction with CHW (%)				
Once	47.8	29.5	52.2	0.87
Twice	26.3	29.9	25.4	
Three times	10.9	17.2	9.4	
Four times	7.7	9.7	7.3	
Five or more times	4.4	2.1	4.8	
Don’t know	2.9	11.5	0.8	
Where talked with CHW (%)				
Home visit	42.4	19.9	47.7	0.002
Health facility	22.4	47.5	16.3	0.02
Village clinic	21.8	22.1	21.7	0.97
Outreach clinic	15.8	8.9	17.5	0.13
Topic discussed with CHW (%)				
Diet during pregnancy	38.6	36.8	39.0	0.90
Plans for delivery	36.1	20.2	39.9	0.09
When to visit ANC	35.4	50.8	31.7	0.03
Signs of a problem with my pregnancy	24.0	21.1	24.7	0.76
Changes to expect in my body	9.3	13.7	8.2	0.34
Medicines that are safe for pregnancy	8.3	7.8	8.4	0.54
Advice given by CHW (%)				
Sleep under ITN	92.0	95.0	91.0	0.35
Take IPTp	28.0	33.9	25.9	0.08
Diet during pregnancy	7.0	4.1	8.0	0.22
Attend ANC regularly	6.3	8.7	5.4	0.34
Signs of problem with pregnancy	1.5	4.2	0.6	< 0.0001
Medicines that are safe for pregnancy	1.4	1.9	1.2	0.56
Changes to expect in my body	0.4	1.3	0.1	0.01

*CHW* Community Health Worker, *ITN* Insecticide-treated Net, ANC antenatal care, *IPTp* intermittent preventive treatment in pregnancy

### ANC attendance

Essentially all women attended at least one ANC visit (99.6%) and half (49.8%) attended four or more ANC visits; 77.5% attended three or more visits in the second and third trimesters based on a review of health passports. According to self-report, only 15.6% of women initiated ANC within the first 12 weeks of pregnancy, as recommended by the WHO. Ninety-four percent of women first disclosed their pregnancy in their first trimester, with their husband being the most common confidant (82.9%) (Table 4).

Women in Nkhata Bay were more likely to receive three or more doses of SP than women in Ntcheu (59.2% vs 41.8% p = 0.0002) (Table 4). Among those who attended at least one ANC visit, the vast majority had positive experiences, with 91.5% rating the care received at ANC as good, very good, or excellent (Table 7).

**Table 7:** Experiences with Antenatal Care (ANC)

Indicator	Overall	Nkhata Bay	Ncheu	p-value
	N = 276	N = 137	N = 139	
Rate ANC (%)				
Excellent	21.0	31.3	15.2	0.01
Very good	41.4	41.4	41.4	
Good	29.1	25.0	31.5	
Fair	4.4	1.3	6.1	
Poor	3.8	0.8	5.6	
Obstacles to getting malaria treatment while pregnant				
No obstacles	35.1	38.1	34.2	0.62
Stock-outs of LA and RDTs at health facilities	15.9	8.2	18.1	0.03
Distance to treatment	15.0	22.9	12.6	0.06
No time to seek care	9.9	10.0	9.9	0.97
Poor quality of care at health facility	8.7	12.5	7.6	0.13
Expense of transport	8.5	12.1	7.4	0.35
Expense of medication	8.4	1.0	10.6	.
Did not want to go alone	4.1	4.2	4.1	0.96
Husband/family does not approve	1.0	4.2	.	.
Few female providers	0.8	3.4	.	.

	N = 368	N = 177	N = 191	
Attended ANC with an escort	57.2	41.6	61.8	0.01

	N = 200	N = 75	N = 125	
People who escorted women to ANC				
Husband/partner	95.3	91.5	96.1	0.06
Mother	2.3	5.5	1.6	
Sister	0.7	2.1	0.4	
Male cousin	0.4	.	0.5	
Friend	1.2	0.4	1.3	
Travel time to ANC (%)				
< 30 min	21.2	25.0	20.1	0.76
30–60 min	14.1	18.1	12.9	
1–2 h	31.5	28.3	32.4	
> 2–3 h	19.1	20.1	18.7	
> 3 + h	14.2	8.5	15.9	

LA Lumefantrine-artemether, first line malaria treatment in Malawi, *RDTs* Rapid Diagnostic Test, *ANC* antenatal care

One-third of women (35.3%) travelled less than 1 h to reach ANC, another one-third (31.5%) travelled from 1 to 2 h, 19.1% travelled 2–3 h, and 14% travelled three hours or more. The majority (86.8%) of the women surveyed reported zero cost associated with traveling to ANC; 78.9% reported that they walked and 8.6% rode a bicycle. The majority of women surveyed (65%) reported at least one obstacle to receiving malaria treatment while pregnant. The most commonly reported obstacles were stock-outs of malaria tests and treatment at health facilities and the distance to treatment (15.9% and 15.0%, respectively).

## Discussion

Although 46% of women interviewed received three or more doses of IPTp, the WHO target of 85% IPTp3 coverage has not yet been achieved in Malawi, highlighting the need for innovative approaches to increase IPTp-SP coverage. The data highlight a need to better understand why a higher percentage of women are not receiving at least three doses of SP, despite 84% attending three or more ANC visits and half attending four or more visits. There is also a need for increased efforts to improve women’s understanding of the dangers of malaria in pregnancy and the benefits of IPTp3+ and early ANC attendance.

Although most women recognized ITNs as a means to prevent malaria in pregnancy, less than one quarter cited IPTp as a method of malaria prevention. Women who cited both ITNs and IPTp as ways of preventing malaria had increased odds of receiving IPTp which approached statistical significance. There were clear regional differences; women in Nkhata bay were more likely to be assisted by a CHW, had better knowledge on the cause and means of preventing malaria, and were more likely to receive IPTp3+ .

Overall, less than half of women surveyed cited malaria as being a serious problem in pregnancy; this has similarly been reported by a number of other studies [15–17]. This lack of awareness may contribute to failure to seek out IPTp. Women’s awareness of the dangers of malaria in pregnancy was lower in Ntcheu than Nkhata Bay, corresponding to lower IPTp3 uptake. Women in Ntcheu reported discussing malaria less often with CHWs than women in Nkhata Bay. The lack of malaria health information shared by CHWs in Ntcheu may be contributing to women’s decreased knowledge about the dangers of malaria in pregnancy, and, consequently, to the lower uptake of IPTp. To improve community-level health education, it is important to provide CHWs with additional education, especially on prevention of malaria in pregnancy.

CHWs are tasked with addressing numerous health issues within communities, and the importance of early and frequent ANC attendance, as well as the dangers of malaria in pregnancy, has not been prioritized. It is anticipated that including discussions on these health topics during every CHW interaction with pregnant women in their communities will increase both early and frequent ANC visits. Including men in health education and messaging related to the importance of early ANC attendance may also increase the number of women attending it the first trimester, as husbands are frequently the first person told about a pregnancy and they can encourage their wives/partners to attend ANC. Male involvement has previously been suggested as a way to encourage earlier attendance at ANC [18–20]. Women who come to ANC without their husbands may face additional barriers, such as being made to wait to be seen until after all the women who came with their husbands or being required to bring a letter from their village chief, which likely contribute to later and less frequent ANC visits (Alinafe Chibwana, pers. commun.).

Counseling on ANC attendance is a critical component of the planned pilot, so that community distribution of IPTp is not perceived as a replacement for an ANC visit. It is particularly important to address the fact that the primary message on malaria prevention women received was to sleep under a bed net, while only a quarter were told by the CHW to take IPTp-SP. Discussing the dangers of malaria in pregnancy and how malaria can be prevented by sleeping under an ITN and especially by receiving regular doses of IPTp-SP should also help to improve uptake of these interventions.

Increased education and community delivery of IPTp-SP are not replacements for regularly scheduled ANC visits, but may be a valuable supplement to these services. Although Malawi has adopted the WHO eight-contact schedule, this does not include monthly visits in early second trimester. Further, many women face barriers to attending ANC. Given the drop off in attendance from three visits (84%) to four or more visits (50% of women) currently, it seems unlikely that a high proportion of women will attend eight ANC visits only as a result of a policy change, without additional changes to the system.

Distribution of SP in the community will help to address the issue of women traveling long distances to attend ANC visits, and allow for more frequent delivery of IPTp. Even with the currently recommended schedule of eight ANC contacts, early in 2nd trimester the visits are widely spaced, thus availability of SP in the community would allow for dosing in between ANC visits. Further, CHWs will provide education on the importance of ANC and IPTp, which is hypothesized to improve the perceived value of those services. CHWs are an important resource within communities and there is great potential for them to improve the health of pregnant women and their infants, if they are empowered to do so.

### Limitations

As with most surveys, there are limitations due to potential recall and social desirability biases. Participants may not have recalled exact dates or interactions with health workers. Further, because the respondent knew that this survey was being administered in preparation for a malaria intervention, it is possible that responses were exaggerated or altered if the respondent perceived that this would increase their chances of receiving a benefit from the subsequent study. It is also possible that women responded with what they perceived were the “correct” responses. In addition, because the survey focused on recently pregnant women and did not include ANC providers, it is not possible to fully understand the causes of the gap between ANC attendance and IPTp coverage. The results presented here may not reflect the situation in other districts in Malawi. However, there is no specific reason to suspect that the opinions of the community about CHWs in other areas would vary substantially.

## Conclusion

Many pregnant women throughout Malawi are not receiving the WHO recommendation of at least 3 doses of IPTp. Lack of understanding of the importance of IPTp-SP and the potential severity of malaria in pregnancy may be additional obstacles to improving coverage. New approaches are needed to help women understand the importance of IPTp, and to improve uptake. In Nkhata Bay, where women were more knowledgeable, IPTp3+ coverage was higher. It is anticipated that by increasing CHWs’ focus on malaria in pregnancy, encouraging routine ANC attendance and IPTp uptake, and providing IPTp-SP in communities, Malawi can make progress towards the goal of 85% of pregnant women receiving three or more doses of SP.

## Data Availability

The datasets used and/or analyzed during the current study are available from the corresponding author on reasonable request.

## Abbreviations

ANC: Antenatal care
CDC: Centers for Disease Control and Prevention
CHW: Community Health Workers
CI: Confidence intervals
cIPTp: Community delivery of intermittent preventive treatment in pregnancy
COMREC: Malawi College of Medicine Research Ethics Committee
EA: Enumeration areas
FANC: Focused antenatal care
IPTp: Intermittent preventive treatment in pregnancy
IPTp-SP: Intermittent preventive treatment in pregnancy with sulfadoxine-pyrimethamine
IPTp2: Two doses of intermittent preventive treatment in pregnancy
IPTp3+: Three or more doses of intermittent preventive treatment in pregnancy
ITN: Insecticide-treated nets
LA: Lumefantrine-artemether, first-line malaria treatment in Malawi
MIP: Malaria in pregnancy
RDT: Rapid diagnostic test
SP: Sulfadoxine-pyrimethamine
WHO: World Health Organization
